# Supplementary material for: Deletion of wfs1 Impairs Oligodendrocyte Precursor Cells Dorsal Distribution and Myelination Through the wfs1-hmgcs1 Axis in Zebrafish
Source: Biology (Basel). 2026 Aug 16;15(16):1402. doi: 10.3390/biology15161402 (PMC13510151; doi:10.3390/biology15161402)
Supplement: Supplementary file 1 [file biology-15-01402-s001.zip › File S1-Transcriptome sequencing data and differentially expressed genes (DEGs) in wfs1-- zebrafish at 5 dpf/1.GO/wfs1KOvsWT/DOWN/wfs1KOvsWT.down_GObar_cc.pdf]

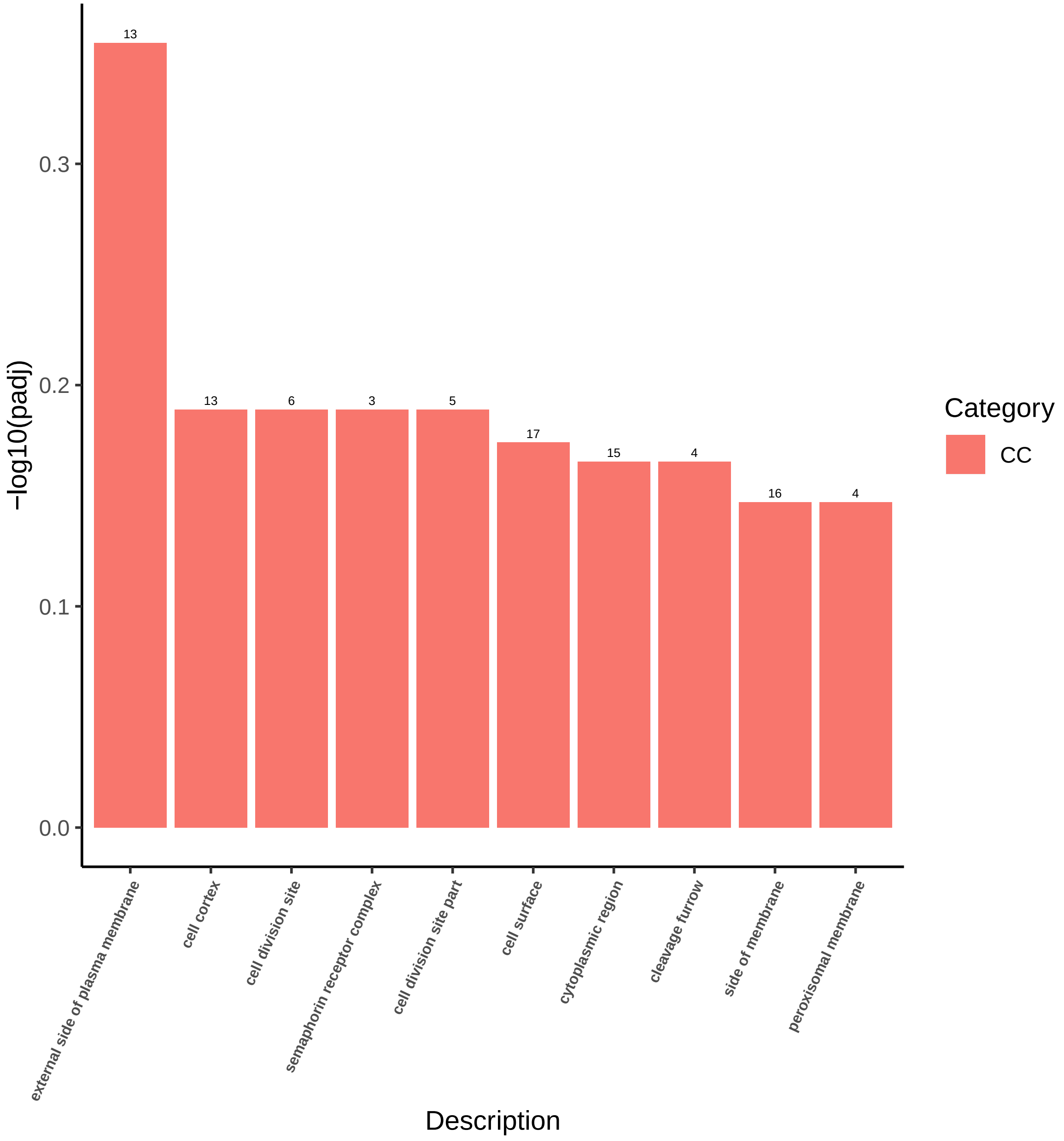
13
13
6
3
5
17
15
4
16
4
0.3
0.2
0.1
0.0
−log10(padj)
external side of plasma membrane
cell cortex
cell division site
semaphorin receptor complex
cell division site part
cell surface
cytoplasmic region
cleavage furrow
side of membrane
peroxisomal membrane
Description
Category
CC
